# *catena*-Poly[[chloridocobalt(II)]-μ-5-(8-quinolyloxymeth­yl)tetra­zolato-κ^4^ *N* ^5^,*O*,*N* ^1^:*N* ^4^]

**DOI:** 10.1107/S1600536808020205

**Published:** 2008-07-09

**Authors:** Guo-Xi Wang, Heng-Yun Ye

**Affiliations:** a Ordered Matter Science Research Center, College of Chemistry and Chemical Engineering, Southeast University, Nanjing 210096, People’s Republic of China

## Abstract

In the title compound, [Co(C_11_H_8_N_5_O)Cl]_*n*_, the Co^II^ atom is penta­coordinated by one O atom and two N atoms from a 5-(8-quinolyloxymeth­yl)tetra­zolate ligand, one N atom from another symmetry-related ligand, and a Cl atom. The coordination geometry can be described as slightly distorted trigonal–bipyramidal. Adjacent Co atoms are connected by the bridging tetra­zole groups into a chain. The dihedral angle between the quinoline and tetra­zole planes is 21.2 (1)°. The structure involves intra- and inter­chain C—H⋯N hydrogen bonds.

## Related literature

For related literature, see: Luo & Ye (2008 ▶); Wang *et al.* (2005 ▶); Wang & Ye (2007 ▶); Xiong *et al.* (2002 ▶).
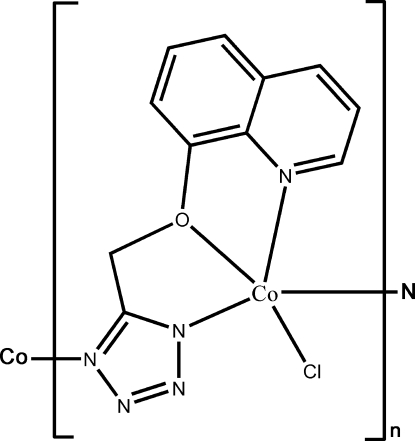


## Experimental

### 

#### Crystal data

[Co(C_11_H_8_N_5_O)Cl]
*M* *_r_* = 320.60
Monoclinic, 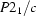

*a* = 7.0289 (11) Å
*b* = 8.4331 (11) Å
*c* = 20.220 (4) Å
β = 96.757 (10)°
*V* = 1190.2 (3) Å^3^
*Z* = 4
Mo *K*α radiation
μ = 1.66 mm^−1^
*T* = 293 (2) K
0.20 × 0.18 × 0.14 mm

#### Data collection

Rigaku SCXmini CCD area-detector diffractometer
Absorption correction: multi-scan (*CrystalClear*; Rigaku, 2005 ▶) *T*_min_ = 0.701, *T*_max_ = 0.798
12192 measured reflections
2846 independent reflections
2385 reflections with *I* > 2σ(*I*)
*R*_int_ = 0.047

#### Refinement

*R*[*F* ^2^ > 2σ(*F* ^2^)] = 0.040
*wR*(*F* ^2^) = 0.101
*S* = 1.08
2846 reflections
172 parameters
H-atom parameters constrained
Δρ_max_ = 0.39 e Å^−3^
Δρ_min_ = −0.37 e Å^−3^

### 

Data collection: *CrystalClear* (Rigaku, 2005 ▶); cell refinement: *CrystalClear*; data reduction: *CrystalClear*; program(s) used to solve structure: *SHELXS97* (Sheldrick, 2008 ▶); program(s) used to refine structure: *SHELXL97* (Sheldrick, 2008 ▶); molecular graphics: *SHELXTL* (Sheldrick, 2008 ▶); software used to prepare material for publication: *SHELXTL*.

## Supplementary Material

Crystal structure: contains datablocks I, global. DOI: 10.1107/S1600536808020205/hy2140sup1.cif

Structure factors: contains datablocks I. DOI: 10.1107/S1600536808020205/hy2140Isup2.hkl

Additional supplementary materials:  crystallographic information; 3D view; checkCIF report

## Figures and Tables

**Table:** 

Co1—N1^i^	2.049 (2)
Co1—N4	2.053 (2)
Co1—N5	2.066 (2)
Co1—Cl1	2.2670 (8)
Co1—O1	2.3979 (18)

**Table:** 

N1^i^—Co1—N4	96.46 (9)
N1^i^—Co1—N5	106.43 (8)
N4—Co1—N5	134.43 (8)
N1^i^—Co1—Cl1	104.93 (7)
N4—Co1—Cl1	106.62 (7)
N5—Co1—Cl1	104.74 (6)
N1^i^—Co1—O1	86.22 (8)
N4—Co1—O1	70.65 (7)
N5—Co1—O1	72.16 (7)
Cl1—Co1—O1	168.82 (6)

Symmetry code: (i) 
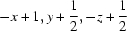
.

**Table 2:** Hydrogen-bond geometry (Å, °)

*D*—H⋯*A*	*D*—H	H⋯*A*	*D*⋯*A*	*D*—H⋯*A*
C2—H2*B*⋯N3^ii^	0.97	2.54	3.292 (4)	135
C8—H8*A*⋯N2^iii^	0.93	2.52	3.398 (4)	157

Symmetry codes: (ii) 
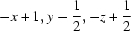
; (iii) 
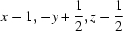
.

**Fig. 1.**
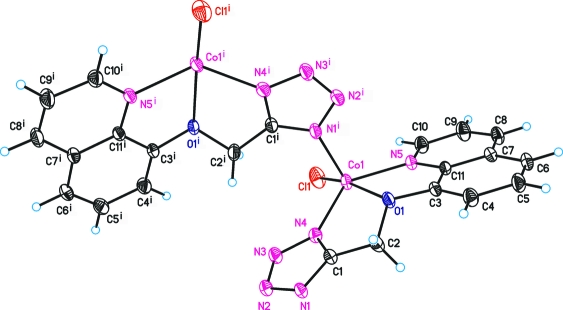
The structure of the title compound. Displacement ellipsoids are drawn at the 30% probability level. [Symmetry code: (i) -x + 1, y + 1/2, -z + 1/2.]

**Fig. 2.**
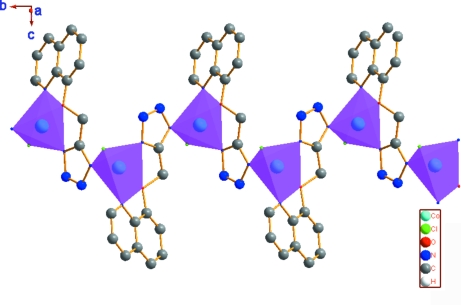
A polyhedral drawing of the chain in the title compound.

**Fig. 3.**
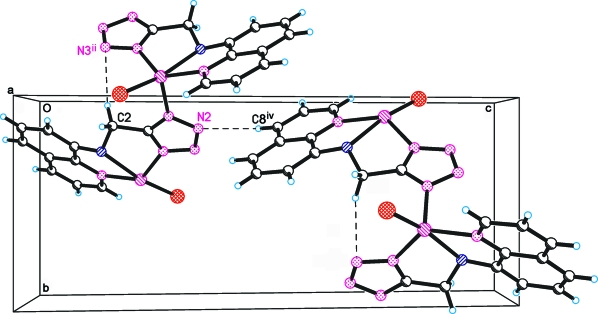
Crystal packing of the title compound viewed along the a-axis. Dashed lines denote hydrogen bonds. [Symmetry codes: (ii) -x + 1, y - 1/2, -z + 1/2; (iv) x + 1, -y + 1/2, z + 1/2.]

## supplementary crystallographic information

### Comment

In the past five years, we have focused on the chemistry of 5-substituted tetrazoles because of their multiple coordination modes to metal ions and the construction of novel metal–organic frameworks. (Wang *et al.*, 2005; Xiong *et al.*, 2002). As part of our on going studies of the chemistry of tetrazoles, we have determined the crystal structure of the title compound.

In the title compound, the Co^II^ atom is penta-coordinated by one O atom and two N atoms from an 8-[(tetrazol-5-yl)methoxy]quinoline ligand, by one N atom from another symmetry-related ligand, and by one terminal Cl atom (Fig. 1). The coordination geometry can be described as slightly distorted trigonal–bipyramidal (Table 1), with three N atoms (N4, N5 and N1^i^) [symmetry code: (i) -*x* + 1, *y* + 1/2, -*z* + 1/2] forming the equatorial palne and the O1 and Cl1 atoms occupying the axial positions. The deviation of the Co1 atom from the equatorial plane is 0.549 (1) Å. The bond angles of N4—Co1—N5, N5—Co1—N1^i^ and N4—Co1—N1^i^ are 134.43 (8), 106.43 (8) and 96.46 (9)°, respectively. Adjacent Co atoms are connected by the bridging tetrazole groups into a chain (Fig. 2). Geometric parameters of the ligand are in normal ranges (Wang & Ye, 2007). The dihedral angle between the quinoline and tetrazole planes is 21.2 (1)°. The structure involves intrachain and interchain C—H···N hydrogen bonds (Fig. 3; Table 2).

### Experimental

8-(1*H*-Tetrazol-5-yl)methoxy)quinoline was synthesized by using a similar procedure described previously by us (Luo & Ye, 2008). A mixture of the ligand (0.045 g, 0.2 mmol), CoCl_2_ (0.026 g, 0.2 mmol) and water (1 ml) was sealed in a glass tube and maintained at 383 K. Purple crystals of the title compound suitable for X-ray ananlysis were obtained after 3 d.

### Refinement

H atoms were positioned geometrically and refined using a riding model, with C—H = 0.93 (aromatic) and 0.97 (CH_2_) Å and with *U*_iso_(H) = 1.2*U*_eq_(C).

### Crystal data

**Table:**

[Co(C_11_H_8_N_5_O)Cl]	*F*_000_ = 644
*M**_r_* = 320.60	*D*_x_ = 1.789 Mg m^−^^3^
Monoclinic, *P*2_1_/*c*	Mo *K*α radiation λ = 0.71073 Å
Hall symbol: -P 2ybc	Cell parameters from 2846 reflections
*a* = 7.0289 (11) Å	θ = 2.6–27.9º
*b* = 8.4331 (11) Å	µ = 1.66 mm^−^^1^
*c* = 20.220 (4) Å	*T* = 293 (2) K
β = 96.757 (10)º	Prism, purple
*V* = 1190.2 (3) Å^3^	0.20 × 0.18 × 0.14 mm
*Z* = 4	

### Data collection

**Table:**

Rigaku SCXmini CCD area-detector diffractometer	2846 independent reflections
Radiation source: fine-focus sealed tube	2385 reflections with *I* > 2σ(*I*)
Monochromator: graphite	*R*_int_ = 0.047
Detector resolution: 13.6612 pixels mm^-1^	θ_max_ = 27.9º
*T* = 293(2) K	θ_min_ = 2.6º
ω scans	*h* = −9→9
Absorption correction: multi-scan(CrystalClear; Rigaku, 2005)	*k* = −11→11
*T*_min_ = 0.701, *T*_max_ = 0.798	*l* = −26→26
12192 measured reflections	

### Refinement

**Table:**

Refinement on *F*^2^	Secondary atom site location: difference Fourier map
Least-squares matrix: full	Hydrogen site location: inferred from neighbouring sites
*R*[*F*^2^ > 2σ(*F*^2^)] = 0.040	H-atom parameters constrained
*wR*(*F*^2^) = 0.101	*w* = 1/[σ^2^(*F*_o_^2^) + (0.0449*P*)^2^ + 0.5457*P*] where *P* = (*F*_o_^2^ + 2*F*_c_^2^)/3
*S* = 1.08	(Δ/σ)_max_ < 0.001
2846 reflections	Δρ_max_ = 0.39 e Å^−^^3^
172 parameters	Δρ_min_ = −0.37 e Å^−^^3^
Primary atom site location: structure-invariant direct methods	Extinction correction: none

### Fractional atomic coordinates and isotropic or equivalent isotropic displacement parameters (Å^2^)

**Table:**

	*x*	*y*	*z*	*U*_iso_*/*U*_eq_	
Co1	0.19156 (5)	0.40223 (4)	0.221382 (16)	0.02932 (13)	
C1	0.5028 (3)	0.1574 (3)	0.24542 (12)	0.0287 (5)	
C2	0.4852 (4)	0.1332 (4)	0.17171 (13)	0.0380 (6)	
H2A	0.6097	0.1391	0.1556	0.046*	
H2B	0.4281	0.0310	0.1596	0.046*	
C3	0.2568 (4)	0.2342 (3)	0.08430 (11)	0.0279 (5)	
C4	0.3194 (4)	0.1528 (4)	0.03257 (13)	0.0376 (6)	
H4A	0.4403	0.1065	0.0375	0.045*	
C5	0.1997 (4)	0.1392 (4)	−0.02832 (13)	0.0404 (7)	
H5A	0.2424	0.0841	−0.0636	0.049*	
C6	0.0216 (4)	0.2065 (3)	−0.03590 (13)	0.0369 (6)	
H6A	−0.0553	0.1978	−0.0764	0.044*	
C7	−0.0466 (4)	0.2890 (3)	0.01735 (12)	0.0303 (5)	
C8	−0.2301 (4)	0.3590 (4)	0.01327 (14)	0.0403 (6)	
H8A	−0.3132	0.3524	−0.0260	0.048*	
C9	−0.2846 (4)	0.4360 (4)	0.06704 (15)	0.0454 (7)	
H9A	−0.4052	0.4825	0.0647	0.054*	
C10	−0.1581 (4)	0.4453 (4)	0.12616 (14)	0.0375 (6)	
H10A	−0.1980	0.4984	0.1624	0.045*	
C11	0.0727 (3)	0.3037 (3)	0.07850 (11)	0.0253 (5)	
N1	0.6256 (3)	0.0865 (3)	0.29043 (10)	0.0303 (5)	
N2	0.5887 (3)	0.1467 (3)	0.34993 (10)	0.0362 (5)	
N3	0.4486 (3)	0.2482 (3)	0.34007 (11)	0.0359 (5)	
N4	0.3906 (3)	0.2574 (3)	0.27357 (10)	0.0309 (5)	
N5	0.0151 (3)	0.3818 (2)	0.13259 (10)	0.0272 (4)	
Cl1	−0.00233 (11)	0.49197 (11)	0.29524 (4)	0.0531 (2)	
O1	0.3637 (3)	0.2596 (2)	0.14477 (8)	0.0324 (4)	

### Atomic displacement parameters (Å^2^)

**Table:**

	*U*^11^	*U*^22^	*U*^33^	*U*^12^	*U*^13^	*U*^23^
Co1	0.0282 (2)	0.0378 (2)	0.02076 (19)	0.00290 (14)	−0.00200 (13)	−0.00415 (14)
C1	0.0287 (12)	0.0331 (13)	0.0232 (12)	0.0014 (10)	−0.0014 (9)	0.0009 (10)
C2	0.0440 (16)	0.0439 (16)	0.0246 (13)	0.0178 (12)	−0.0024 (11)	0.0015 (12)
C3	0.0333 (13)	0.0308 (12)	0.0183 (11)	0.0017 (10)	−0.0019 (9)	0.0010 (10)
C4	0.0387 (15)	0.0451 (15)	0.0286 (13)	0.0113 (12)	0.0018 (11)	−0.0043 (12)
C5	0.0511 (17)	0.0481 (16)	0.0221 (13)	0.0060 (13)	0.0041 (11)	−0.0077 (12)
C6	0.0450 (15)	0.0441 (16)	0.0197 (12)	0.0004 (12)	−0.0036 (11)	−0.0027 (11)
C7	0.0346 (13)	0.0333 (13)	0.0217 (12)	−0.0004 (10)	−0.0026 (10)	0.0012 (10)
C8	0.0356 (15)	0.0532 (17)	0.0287 (14)	0.0039 (12)	−0.0099 (11)	−0.0020 (13)
C9	0.0330 (14)	0.063 (2)	0.0376 (16)	0.0142 (13)	−0.0065 (12)	−0.0070 (14)
C10	0.0343 (14)	0.0457 (16)	0.0312 (14)	0.0101 (12)	−0.0011 (11)	−0.0056 (12)
C11	0.0283 (12)	0.0278 (12)	0.0189 (11)	−0.0020 (9)	−0.0010 (9)	0.0001 (9)
N1	0.0345 (11)	0.0343 (11)	0.0204 (10)	−0.0012 (9)	−0.0031 (8)	0.0026 (8)
N2	0.0473 (14)	0.0382 (12)	0.0214 (11)	−0.0021 (10)	−0.0027 (9)	0.0010 (9)
N3	0.0448 (13)	0.0405 (13)	0.0216 (10)	−0.0018 (10)	0.0005 (9)	0.0005 (9)
N4	0.0357 (12)	0.0329 (11)	0.0235 (10)	−0.0008 (9)	0.0012 (9)	−0.0010 (9)
N5	0.0286 (10)	0.0318 (11)	0.0205 (10)	0.0008 (8)	−0.0008 (8)	−0.0019 (8)
Cl1	0.0478 (4)	0.0800 (6)	0.0326 (4)	0.0160 (4)	0.0090 (3)	−0.0097 (4)
O1	0.0347 (10)	0.0374 (10)	0.0227 (9)	0.0098 (7)	−0.0072 (7)	−0.0021 (8)

### Geometric parameters (Å, °)

**Table:**

Co1—N1^i^	2.049 (2)	C5—H5A	0.9300
Co1—N4	2.053 (2)	C6—C7	1.413 (4)
Co1—N5	2.066 (2)	C6—H6A	0.9300
Co1—Cl1	2.2670 (8)	C7—C8	1.412 (4)
Co1—O1	2.3979 (18)	C7—C11	1.415 (3)
C1—N1	1.321 (3)	C8—C9	1.360 (4)
C1—N4	1.327 (3)	C8—H8A	0.9300
C1—C2	1.495 (3)	C9—C10	1.406 (4)
C2—O1	1.433 (3)	C9—H9A	0.9300
C2—H2A	0.9700	C10—N5	1.322 (3)
C2—H2B	0.9700	C10—H10A	0.9300
C3—C4	1.366 (3)	C11—N5	1.378 (3)
C3—O1	1.375 (3)	N1—N2	1.359 (3)
C3—C11	1.413 (3)	N1—Co1^ii^	2.049 (2)
C4—C5	1.412 (4)	N2—N3	1.302 (3)
C4—H4A	0.9300	N3—N4	1.361 (3)
C5—C6	1.366 (4)		
			
N1^i^—Co1—N4	96.46 (9)	C7—C6—H6A	119.7
N1^i^—Co1—N5	106.43 (8)	C8—C7—C6	123.4 (2)
N4—Co1—N5	134.43 (8)	C8—C7—C11	117.3 (2)
N1^i^—Co1—Cl1	104.93 (7)	C6—C7—C11	119.3 (2)
N4—Co1—Cl1	106.62 (7)	C9—C8—C7	119.6 (2)
N5—Co1—Cl1	104.74 (6)	C9—C8—H8A	120.2
N1^i^—Co1—O1	86.22 (8)	C7—C8—H8A	120.2
N4—Co1—O1	70.65 (7)	C8—C9—C10	119.8 (3)
N5—Co1—O1	72.16 (7)	C8—C9—H9A	120.1
Cl1—Co1—O1	168.82 (6)	C10—C9—H9A	120.1
N1—C1—N4	111.4 (2)	N5—C10—C9	123.0 (3)
N1—C1—C2	126.6 (2)	N5—C10—H10A	118.5
N4—C1—C2	122.1 (2)	C9—C10—H10A	118.5
O1—C2—C1	104.7 (2)	N5—C11—C3	119.0 (2)
O1—C2—H2A	110.8	N5—C11—C7	122.4 (2)
C1—C2—H2A	110.8	C3—C11—C7	118.6 (2)
O1—C2—H2B	110.8	C1—N1—N2	105.3 (2)
C1—C2—H2B	110.8	C1—N1—Co1^ii^	129.11 (17)
H2A—C2—H2B	108.9	N2—N1—Co1^ii^	125.07 (16)
C4—C3—O1	124.6 (2)	N3—N2—N1	109.3 (2)
C4—C3—C11	121.3 (2)	N2—N3—N4	108.8 (2)
O1—C3—C11	114.1 (2)	C1—N4—N3	105.3 (2)
C3—C4—C5	119.7 (3)	C1—N4—Co1	124.09 (17)
C3—C4—H4A	120.1	N3—N4—Co1	130.42 (17)
C5—C4—H4A	120.1	C10—N5—C11	117.9 (2)
C6—C5—C4	120.6 (2)	C10—N5—Co1	120.22 (17)
C6—C5—H5A	119.7	C11—N5—Co1	121.84 (16)
C4—C5—H5A	119.7	C3—O1—C2	117.5 (2)
C5—C6—C7	120.5 (2)	C3—O1—Co1	112.87 (14)
C5—C6—H6A	119.7	C2—O1—Co1	116.81 (15)

Symmetry codes: (i) −*x*+1, *y*+1/2, −*z*+1/2; (ii) −*x*+1, *y*−1/2, −*z*+1/2.

### Hydrogen-bond geometry (Å, °)

**Table:**

*D*—H···*A*	*D*—H	H···*A*	*D*···*A*	*D*—H···*A*
C2—H2B···N3^ii^	0.97	2.54	3.292 (4)	135
C8—H8A···N2^iii^	0.93	2.52	3.398 (4)	157

Symmetry codes: (ii) −*x*+1, *y*−1/2, −*z*+1/2; (iii) *x*−1, −*y*+1/2, *z*−1/2.

## References

[bb1] Luo, H.-Z. & Ye, H.-Y. (2008). *Acta Cryst.* E**64**, o136. 10.1107/S1600536807062733 PMC2915205 21200700

[bb2] Rigaku (2005). *CrystalClear* Rigaku Corporation, Tokyo, Japan.

[bb3] Sheldrick, G. M. (2008). *Acta Cryst.* A**64**, 112–122. 10.1107/S0108767307043930 18156677

[bb4] Wang, X.-S., Tang, Y.-Z., Huang, X.-F., Qu, Z.-R., Che, C.-M., Chan, C. W. H. & Xiong, R.-G. (2005). *Inorg. Chem.* **44**, 5278–5285. 10.1021/ic050354x 16022526

[bb5] Wang, G.-X. & Ye, H.-Y. (2007). *Acta Cryst.* E**63**, o4410.

[bb6] Xiong, R.-G., Xue, X., Zhao, H., You, X.-Z., Abrahams, B. F. & Xue, Z.-L. (2002). *Angew. Chem. Int. Ed.* **41**, 3800–3803. 10.1002/1521-3773(20021018)41:20<3800::AID-ANIE3800>3.0.CO;2-3 12386852

